# Mass spectrometry-based proteomic profiling of extracellular vesicle proteins in diabetic and non-diabetic ischemic stroke patients: a case-control study

**DOI:** 10.3389/fmolb.2024.1387859

**Published:** 2024-06-14

**Authors:** Shahnaz Qadri, Muhamad U. Sohail, Naveed Akhtar, Ghulam Jeelani Pir, Ghada Yousif, Sajitha V. Pananchikkal, Muna Al-Noubi, Sunkyu Choi, Ashfaq Shuaib, Yousef Haik, Aijaz Parray, Frank Schmidt

**Affiliations:** ^1^ Department of Pharmaceutical Sciences, Irma Lerma Rangel School of Pharmacy, Texas A&M University, Kingsville, TX, United States; ^2^ Sustainability Division, College of Science and Engineering, Hamad Bin Khalifa University, Doha, Qatar; ^3^ Proteomics Core, Weill Cornell Medicine, Doha, Qatar; ^4^ The Neuroscience Institute, Academic Health System, Hamad Medical Corporation, Doha, Qatar; ^5^ College of Health and Life Sciences, Hamad Bin Khalifa University, Doha, Qatar; ^6^ Department of Medicine, University of Alberta, Edmonton, AB, Canada; ^7^ Department of Mechanical and Nuclear Engineering, University of Sharjah, Sharjah, United Arab Emirates

**Keywords:** extracellular vesicles (EVs), diabetes, stroke, exosomes, mass spectrometry, blood coagulation, tissue injury, complement system

## Abstract

Acute ischemic stroke is the most common cause of neurologic dysfunction caused by focal brain ischemia and tissue injury. Diabetes is a major risk factor of stroke, exacerbating disease management and prognosis. Therefore, discovering new diagnostic markers and therapeutic targets is critical for stroke prevention and treatment. Extracellular vesicles (EVs), with their distinctive properties, have emerged as promising candidates for biomarker discovery and therapeutic application. This case-control study utilized mass spectrometry-based proteomics to compare EVs from non-diabetic stroke (nDS = 14), diabetic stroke (DS = 13), and healthy control (HC = 12) subjects. Among 1288 identified proteins, 387 were statistically compared. Statistical comparisons using a general linear model (log2 foldchange ≥0.58 and FDR-p≤0.05) were performed for nDS vs HC, DS vs HC, and DS vs nDS. DS vs HC and DS vs nDS comparisons produced 123 and 149 differentially expressed proteins, respectively. Fibrinogen gamma chain (FIBG), Fibrinogen beta chain (FIBB), Tetratricopeptide repeat protein 16 (TTC16), Proline rich 14-like (PR14L), Inhibitor of nuclear factor kappa-B kinase subunit epsilon (IKKE), Biorientation of chromosomes in cell division protein 1-like 1 (BD1L1), and protein PR14L exhibited significant differences in the DS group. The pathway analysis revealed that the complement system pathways were activated, and blood coagulation and neuroprotection were inhibited in the DS group (z-score ≥2; *p* ≤ 0.05). These findings underscore the potential of EVs proteomics in identifying biomarkers for stroke management and prevention, warranting further clinical investigation.

## 1 Introduction

Acute ischemic stroke is a cerebrovascular disease with a high risk of neurological disability and mortality. Vascular diseases are the leading cause of death (18.6 million in 2019), with stroke ranking second in the category (Roth et al., 2020). Stroke currently accounts for around 3%–4% of total healthcare expenditures in Western countries (Struijs et al., 2006). In the United States, the average lifetime cost of an ischemic stroke, including hospitalization, rehabilitation, and follow-up, is estimated at $140,048 per person (Johnson et al., 2016). In Qatar, stroke incidence and mortality rates are comparatively low, with documented figures of 58 and 9.17 cases per 100,000 inhabitants per year, respectively (Jallow et al., 2019). Furthermore, the prevailing comorbidities among Qatari stroke patients are hypertension (82.7%) and diabetes (71.6%) (Imam et al., 2020).

Diabetes affects nearly 33% of stroke patients and strongly predicts ischemic stroke (Lau et al., 2019). Diabetes can cause several microvascular and macrovascular changes that frequently have substantial clinical repercussions, including stroke (Venkat et al., 2018). Diabetes promotes systemic inflammation, vascular endothelial dysfunction, basement membrane thickening, and arterial stiffness (Maida et al., 2022). Diabetes inhibits nitric oxide mediated vasodilation, which can lead to endothelial dysfunction and atherosclerosis (Amelia and Harahap, 2019). Consistent with this, a study highlighted distinctively regulated circulating miRNA in stroke patients with diabetes. Some of these miRNAs are implicated in pathways related to vasculature and disrupted homeostasis (Toor et al., 2022). Stroke patients with persistent hyperglycemia have a poor prognosis and an increased post-stroke mortality rate (Hou et al., 2021). Recently, advances in stroke management and treatment have decreased mortality. However, the aging population and rise in diabetes have significantly increased the lifetime risk of stroke. Diabetic stroke (DS) patients are more likely to struggle for basic self-care, suffer participation restrictions in post-stroke life, and are more likely to have recurrent stroke attacks and death due to stroke (Billinger et al., 2014; Cowie et al., 2018).

The speed with which ischemic stroke causes neuronal damage and mortality limits the time window for successful medical intervention. Treatment with an intravenous tissue-type plasminogen activator (tPA) is the only effective treatment in patients with acute ischemic stroke (Ehrlich et al., 2019). However, in many clinical settings, the use of tPA in DS is either not beneficial or not recommended (Jiang et al., 2021). Therefore, early diagnosis and novel therapeutics at sub-acute time points are essential for DS patients. Recent advances in stem cell and EVs therapeutics have been the cornerstone of novel approaches that may enhance the natural healing of ischemic brain tissue (Zhang et al., 2019; Kalluri and LeBleu, 2020).

Exosomes are a subclass of extracellular vesicles that range in diameter from 30 to 150 nm, mediate intercellular communication and transport metabolites (Guay and Regazzi, 2017). Considering exosomes low biodegradability and natural ability to cross the BBB, several studies have focused on their therapeutic application (Khan et al., 2021). The application of exosomes to discover biomarkers and as therapeutic vehicles put them at the forefront of clinical success for various diseases (Lee et al., 2022). For example, Khan et al. (2021) reviewed the application of native and bioengineered EVs for ischemic stroke therapy, reporting positive effects on brain injury and neuron regeneration. An ongoing clinical trial (NCT03384433) for acute ischemic stroke evaluates the efficacy and safety of exosomes derived from allogenic mesenchymal stem cells.

Researching EVs in DS patients is particularly important as these people suffer the most severe effects and have fewer therapeutic choices. Although research on EVs in various cerebrovascular and neurodegeneration diseases is progressing, a more profound comprehension of EV contents is essential for their best clinical use. The present study uses high throughput mass spectrometry to investigate EV proteomics in DS and non-diabetic stroke (nDS) patients. The study’s objective is to find novel biomarkers that could aid in the early detection and management of DS. This study observed more than 1,288 EV proteins in the cohort, of which 387 are used for statistical comparison and pathways analysis.

## 2 Methods and materials

### 2.1 Study participants

The current study was approved by Hamad Medical Corporation IRB (MRC-03-19-020). The patients were admitted to the hospital within 24 h of acute ischemic stroke. After obtaining informed consent, the patient’s demographics were recorded in an electronic case record form. The initial clinical examination of the participants was completed within 24–48 h from the onset of symptoms. All patients had a brain MRI to confirm the location of the stroke. The clinical diagnosis, risk factors, comorbidities, and treatment course were recorded. 27 stroke patients were included in this study (median age = 48 years; gender = 25 males and two females). The stroke patients were divided into two clinical subgroups based on their HbA1c status DS group; HbA1c ≥ 6; *n* = 13) and nDS group; HbA1c ˂6; *n* = 14). In addition, samples were also obtained from 12 healthy controls (median age 46 years; gender = 8 males and four females) with no previous history of stroke. Blood samples were collected in plain tubes and allowed to clot for 30 min at room temperature and later centrifuged at 1,500 *g* for 10 min to separate serum. All serum samples were stored at −80°C until the EV isolation and mass spectrometry analysis were performed.

### 2.2 EVs isolation by ultracentrifugation

Equal volumes of serum (200 µL) diluted in PBS containing protease inhibitors from all the subjects were differentially centrifuged at 3,000 *g* for 15 min to remove cell debris and larger vesicles. The supernatants were transferred to sterile microcentrifuge tubes and were centrifuged at 20,000 *g* for 20 min at 4°C (Eppendorf Centrifuge 5424R, Hamburg, Germany) to isolate the microparticles. The microparticle-free serum (MPS) was diluted in PBS (pH 7.4). The MPS was used for the isolation of EVs by ultracentrifugation (Hitachi CP100NX, Tokyo, Japan) at 100,000 *g* for 120 min at 4°C. The supernatants were transferred to separate vials and pellets were resuspended in 1 mL PBS. The ultracentrifugation procedure was again repeated in similar conditions. The final pellets obtained at 100,000 *g* were resuspended in 30 µL of PBS and stored at −20°C.

### 2.3 EVs validation by CD63 and CD81 western blot

Western blot was performed to characterize CD63 and CD81 EV markers in the isolated samples. Equal volumes (7 µL) of EVs from each sample were lysed in 4x Laemmli buffer (BioRad, Hercules, CA, United States) under reducing conditions with 5 mM of 2-mercapto-ethanol, followed by sample denaturation at 95°C for 10 min. Western blot was performed with 0.2 µm polyvinylidene difluoride (PVDF) membranes. The membrane was blocked with clear milk-blocking buffer prepared in tris buffer saline with tween (TBST; 0.1%) on a rocker in the cold room for 1 h. The blot was then exposed to CD63 and CD81 primary antibodies (Abcam, Cambridge, MA, United States) at 4°C overnight. On the following day, the blots were incubated with the corresponding secondary antibodies (goat anti-mouse-HRP; 1:1,000) for 1 h. After washing, the membranes were visualized by Clarity Western ECL substrate (Bio-Rad) and images were acquired using the Bio-Rad Chemidoc MP Imaging system.

### 2.4 Physical characterization of extracellular vesicles using DLS and TEM

The morphology of EVs was characterized by Transmission Electron Microscopy (TEM) and Dynamic Light Scattering (DLS) following protocols described in our previous studies (Akbar et al., 2022). For TEM, 5–10 µL of EV samples were placed on copper grids with 300 mesh holey-carbon film (TEM-CF300CU50; Sigma-Aldrich) and incubated at room temperature for 15 min. The adsorbed EVs on the TEM grid were fixed by adding a drop (5–10 µL) of 4% of formaldehyde and 2% glutaraldehyde solutions. The grid was washed three times with PBS. Following washing, 5 µL 0.1 M sodium cacodylate buffer was added for 5 min as a negative stain and washed three times again using ultrapure deionized water. Later, 0.2% uranyl acetate (filtered by a 0.2 µm syringe filter) was added to the TEM grid and incubated for 5 min. The grid was washed 5 times with ultrapure distilled water. Sample dehydration was performed with gradient ethanol series twice, incubation starting from 50%, 70%, 80%, and 90% for 5 min each and 100% anhydrous ethanol for 10 min. Finally, the TEM grids were air-dried for 5 min and stored in a vacuum desiccator. Next, the EVs on TEM grids were analyzed under TEM (FEI, Talos F200X).

The hydrodynamic size of EVs was estimated using a Zeta-sizer nano ZSP (Malvern instruments, UK). Briefly, EVs were diluted in 1 X PBS (1:1 ratio), and 10 µL of the samples was transferred to a Quartz microcuvette (ZEN00400). DLS measurements were conducted at 24°C operating at 633 nm, and the back-scattered light was recorded at an angle of 175°. The sample was allowed to equilibrate for 2 min before each measurement. The light scattering was recorded for 200 s with three replicate measurements. Using the Malvern Instruments software DLS signal intensity was transformed to volume distribution, assuming a spherical shape of the EVs.

### 2.5 Pre-digest SDS-PAGE

Invitrogen NuPAGE 4%–12% bis-tris polyacrylamide precast gels (Invitrogen, United States) were used for optimal separation of the proteins and sample quality check using standard protocols (Looze et al., 2009). Samples were prepared by dissolving 10 μL of EVs, 5 μL of NuPAGE LDS sample buffer (4x), 1 μL of dithiothreitol (DTT) (10X), and 6.5 μL of deionized water. The running buffer was composed of 50 mL of NuPAGE 20X MES dissolved in 950 mL of deionized water to prepare 1X SDS.

### 2.6 EV preparation for mass spectrometry

10 μg of protein were used for in-gel digest. Samples were run in NuPAGE 4%–12% Bis-Tris polyacrylamide for 10 min to get one big band. Gels were washed with deionized water for 15 min on the shaker. The big band per lane was cut into small equivalent blocks and transferred to 1.5 mL tubes. For digestion, 12.5 ng/μL of trypsin in 25 mM ammonium bicarbonate (ABC) was used for overnight sample incubated at 37°C. The next day, gel blocks were centrifuged for 1 min at 15,000 *g* and supernatants were transferred into fresh tubes. Later, gel blocks were covered with 30% acetonitrile (ACN) (Thermo Fisher Scientific, United States) and 3% trifluoroacetic acid (TFA) (Sigma-Aldrich, United States) for 10 min, and 100% ACN for 10 min. Finally, the supernatants were concentrated using speedvac at 37°C.

### 2.7 Mass spectrometry measurements

LC-MS/MS analysis was conducted on a Q-Exactive HFX mass spectrometer with a nano-flow chromatographic system (Easy nLC-II 1200, Thermo Scientific). Samples were injected into Acclaim™ PepMap™ 100 Nano-Trap column (particle size 5 μm, 100Å, I.D.100, length 2 cm, Thermo Scientific). Samples were eluted into EASY-spray C18 LC column (particle size 3 μm, 100Å, I.D.50, length 15 cm, Thermo ScientificTM) and separated by a 120-min gradient from buffer A (0.5% formic acid in distilled water to buffer B (80% ACN, 0.5% formic acid (FA). Ionized peptides were introduced into the mass spectrometer by EASY-Spray™ Source (Thermo ScientificTM) at 40°C column temperature. Full scans were acquired at a resolution of 70,000 (*m/z* 300). Data-dependent analysis (DDA) was performed on the 15 most intense ions detected in the full scan. Each LC-MS/MS sample was analyzed in triplicates.

### 2.8 MS data analysis

Raw data were processed with the Genedata Refiner MS software (v13.0.1, Genedata, Basel, Switzerland). MS1 data were aligned between the runs, and MS2 data identifications were transferred to missing values. Processed MS2 spectra were used for identification. The FASTA database search was performed with the MASCOT search algorithm v2.6 against a UniProt/SwissProt database (2019) and limited to human entries while ensuring an adjusted FDR of <1%. The precursor tolerance was set to 20 ppm and MS2 tolerance to 0.05 Da. Only proteins detected with two unique peptides were considered for further quantitative analysis.

### 2.9 Statistical analysis

All statistical analyses were performed using R 4.2.2. Group comparisons were made, and volcano plots were constructed using a general linear model (glmQLFit) in the edgeR package (version 3.14.0). Pairwise contrasts (log2 foldchange (log2FC)) and Benjamini–Hochberg (BH) *p* = 0.05 were used for the analysis. Clinical parameters, including age, gender, BMI, statin usage, and anticoagulant usage, were utilized as covariates for glmQLFit analysis. Volcano plots were constructed for nDS vs HC (Figure 3), DS vs HC (Figure 4), and DS vs nDS (Figure 5) comparisons. PCA plot was constructed using the PCA tools package in R. Glucose, insulin, HbA1c, blood pressure, and lipid profile were compared among clinical parameters using either T-tests or ANOVA.

### 2.10 g: profiler enrichment and IPA pathways analysis

The observed proteins were analysed using the g:Profiler, a web server for functional enrichment analysis. *p*-values were calculated and corrected with BH. Only hits from GO:CC and REAC with a cut-off *p* < 0.01 were allowed.

Differentially expressed proteins (nDS vs HC = 4; DS vs HC = 123, and DS vs nDS = 149) were further processed using the Ingenuity Pathway Analysis (IPA) core analysis for functional pathway annotation. The IPA canonical pathway predicted activation (z-score >2) and inhibition (z-score ≤ −2) of specific pathways using the submitted protein list and log2FC values.

## 3 Results

### 3.1 Study cohort

13 DS and 14 nDS, along with 12 HC subjects, were included in this study. Stroke risk factors in the DS group were hypertension (85%) and dyslipidemia (100%). 15% of DS patients were taking anticoagulants, 77% were taking antihypertension medications, and 100% were taking antiplatelets and statins. As per TOAST classification (Adams et al., 1993), 77% of the DS patients had small vessel disease (SVD), 15% had a stroke of other determined etiology (SDO), and 7% had intracranial hemorrhage (ICH). In the nDS group, stroke risk factors included hypertension (71%), dyslipidemia (91%), and atrial fibrillation (AF) (7%). 14% of nDS patients took anticoagulants, 71% antihypertension, 85% antiplatelets, and 93% statins. According to the TOAST classification, 50% of the nDS patients had SVD, 28.5% had LVD, 7% had a cardioembolic stroke, 7% had SDO, and 7% had ICH. Table 1 summarizes clinical characteristics of the patients.

**TABLE 1:** Clinical characteristics of the healthy, diabetic stroke, and non-diabetic stroke patients.

Study population characteristics	Healthy controls (HC)	Diabetic stroke (DS)	Non-diabetic stroke (nDS)	*p*-value*
Cohort size	12	13	14	--
Gender	8 M + 4 F	12 M + 1 F	13 M + 1 F	--
Age (years) (median (IQR))	46 (13)	53 (13)	45 (16)	--
BMI (mean ± SD)	24.48^a^ ± 1.51	27.08^b^ ± 4.43	27.92^b^ ± 4.65	0.08
Ethnicity	Arab: 5, CAU:1 SA: 6	Arab:2, CAU:1, FE:1, SA:9	African: 1, Arab:3, FE:5, SA:5	--
Admission NIHSS (mean ± SD)	--	4.30 ± 1.75	5.64 ± 3.75	--
Systolic Blood Pressure (mean ± SD)	118.7^a^ ± 10.16	159.15^b^ ± 27.65	154.14^b^ ± 36.59	0.001
Diastolic Blood Pressure (mean ± SD)	76.7^a^ ± 8.65	94.31^b^ ± 12.12	102.92^b^ ± 27.07	0.0025
Glucose conc. (mmol/L) (mean ± SD)	4.45^a^ ± 0.64	11.26^b^ ± 3.43	6.46^a^ ± 1.74	0.0001
HbA1c (mmol/mol) (mean ± SD)	5.41^a^ ± 0.36	9.58^b^ ± 1.66	5.42^a^ ± 0.54	0.0001
Insulin (mIU/L) (mean ± SD)	--	50.83 ± 32.69	26.62 ± 16.27	0.02093
Cholesterol (mmol/L) (mean ± SD)	5.15 ± 0.98	5.28 ± 1.49	5.1 ± 1.08	0.939
HDL-Cholesterol (mmol/L) (mean ± SD)	1.08^b^ ± 0.24	0.89^a^ ± 0.10	1.05^b^ ± 0.20	0.0303
LDL-Cholesterol (mmol/L) (mean ± SD)	3.377 ± 0.28	3.44 ± 1.46	3.38 ± 0.61	0.834
Triglycerides (mmol/L) (mean ± SD)	1.79 ± 1.22	1.56 ± 0.68	1.57 ± 1.07	0.334
Homocysteine (mcmol/L) (mean ± SD)	--	11.29 ± 5.34	11.27 ± 5.69	0.0641
Smokers (% of population)	41.60%	76.92%	50.00%	--

^*^-value is calculated using ANOVA, or *t*-test. Superscripted alphabets (a) and (b) represent groups with significant differences using ANOVA or *t*-test.

### 3.2 Characterization of EVs

**FIGURE 1:**
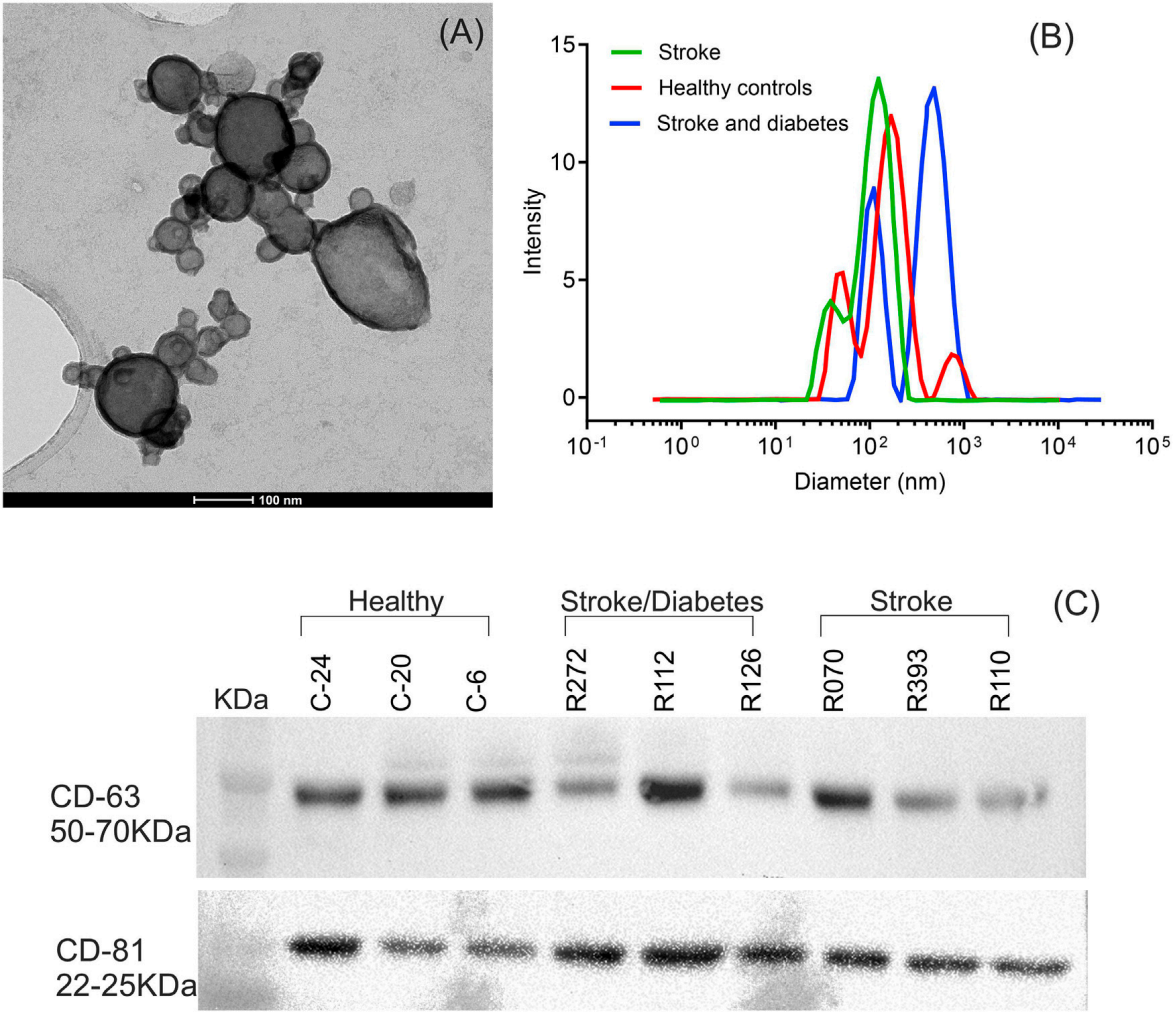
Characterization of isolated serum EVs. **(A)** Transmission electron microscope imaging (TEM) of EVs for particle morphology and size. A representative TEM image of EVs demonstrating membrane-bound vesicles of size ranging from 30 nm to 200 nm. **(B)** Hydrodynamic size distribution of isolated EVs determined by dynamic light scattering (DLS). A representative DLS graph for measurement of EVs. **(C)** Western blot analysis of the EV protein marker CD63 and CD81.

The characterization of EVs by TEM showed that EVs were spherical or oval with an average diameter of 90 nm and sizes ranging from 30 nm to 200 nm. Figure 1A depicts a representative TEM image from a healthy control patient. No differences in EV morphology or sizes were found between the HC, DS, and nDS groups.

Figure 1B depicts the hydrodynamic sizes of EVs from all three groups. DLS size measurements revealed a wide range of EV sizes. The z-average (d.nm) hydrodynamic diameters of EVs from the HC, DS, and nDS were 110 nm, 118.6 nm, and 122 nm, respectively.

Western blot analysis of the endosome-specific tetraspanin CD63 and CD81 was performed to determine the endosomal origin of the EVs. The CD63 and CD81 were seen as band between 50–70 KDa and 22–25 KDa respectively. Figure 1C highlights that CD63 and CD81 were expressed in our samples, inferring that the EV isolation procedure was successful.

In the next step, we aimed to characterize and compare EVs proteome with the serum and EV-free serum samples. Mass spectrometry was employed to analyze four random samples each of EV, serum, and EV-free serum. The PCoA plot analysis demonstrated distinct clustering of EV samples compared to serum and EV-free samples. EV samples formed a distinct cluster, separate from the clustered grouping of serum and EV-free serum samples (Supplementary Figure S1A). This suggests that the proteome of EVs differs from that of serum. Heatmap analysis similarly indicated that proteins elevated in EVs exhibit lower levels in serum samples and *vice versa* (Supplementary Figure S1B). The proteins that were found to be higher in EVs were cross-referenced with the ExoCarta database (http://www.exocarta.org/), an exosome database that provides information on the contents identified in exosomes across multiple organisms. Few of the proteins that exhibited comparatively higher levels in EVs are enlisted in Supplementary Figure S1C. Additionally, proteins highlighted in red correspond to entries documented in the ExoCarta database. Finally, it was also observed that enlisted proteins are interconnected as revealed by protein-protein interaction networks functional enrichment analysis (Supplementary Figure S1D) (https://string-db.org/).

### 3.3 Mass spectrometry analysis

**FIGURE 2:**
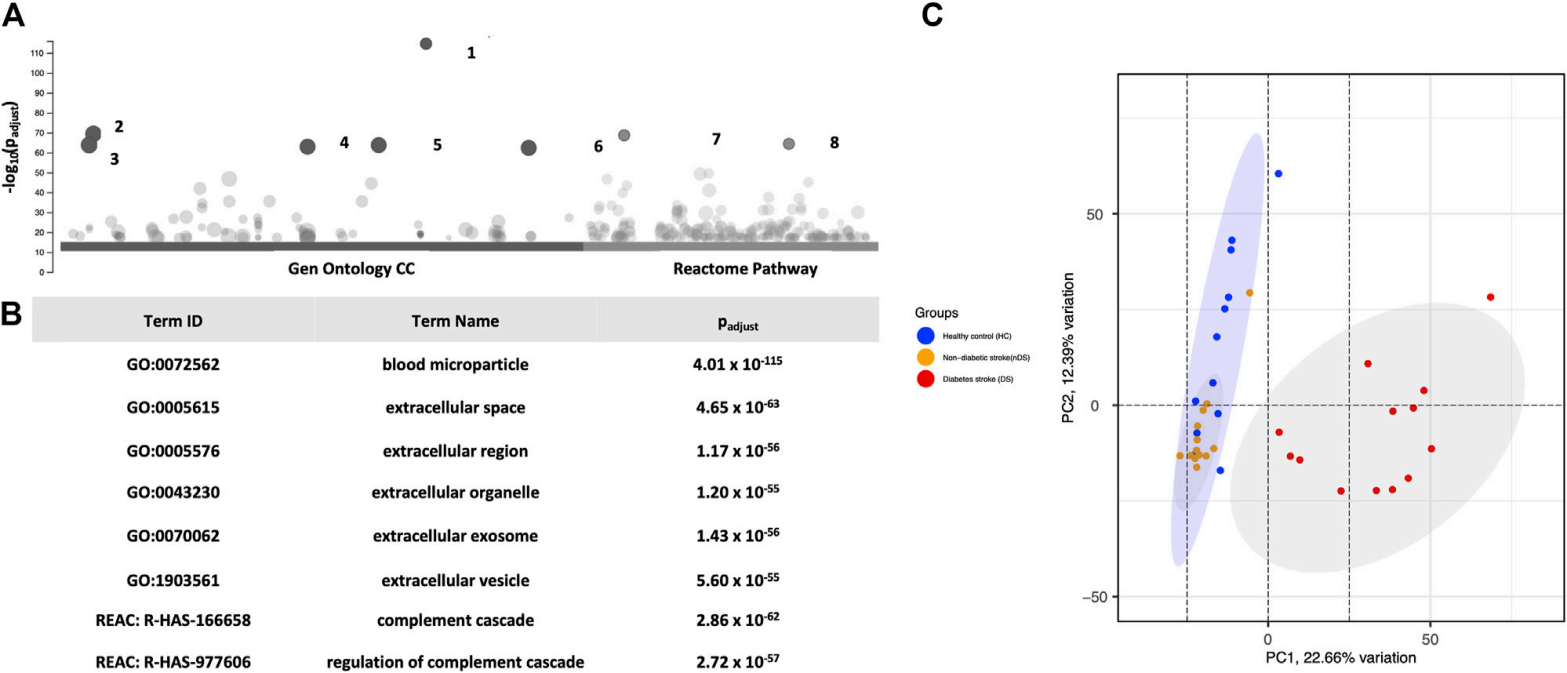
**(A)** Presents **(G)**profiler enrichment analysis of CC Gene Ontology and the REAC DB of all 387 proteins. **(B)** The table highlights the top six Gen Ontology and two REAC hits. PCA Analysis. **(C)** Distribution of the exosomal proteomes in a PCA graph. 2D format of PCA plot ordination similarity analysis of samples using 387 proteins. All dots are plotted against x- and *y*-axes of coordination. The analysis shows that diabetic stroke (red dots) samples clustered separately from the other two groups in the first component. Healthy control (blue dots) and non-diabetic stroke (orange dots) samples mostly overlap with slight divergence on the second PCA component.

The raw data of LC‐MS/MS was processed in REFINER MS^®^ 7.5 software (Genedata, Switzerland). In total, 1,288 proteins were identified in the EVs. G:profiler enrichment analysis for the cellular compartment (GO:CC) revealed that many proteins belonged to extracellular vesicles. For example, blood microparticles (GO:0072562) and EVs (GO:1903561) enlisted most of the observed proteins at a significantly high *p*-value (P_adj_ ≤ 5.6 × 10^−55^). Furthermore, the g:profiler REAC analysis mapped EV proteins associated with the complement system (REAC:R-HAS-166658 and REAC:R-HAS-977606). Figures 2A, B show the top six GO:CC and two GO:REAC hits with significant *p*-values for the annotated functions. The analysis confirmed the EV origin of the proteins.

In the next step, proteins (n = 387) with more than two unique peptides were used for statistical comparison. A PCA plot was constructed to compare the study groups (Figure 2C). Changes in the DS group proteome was highlighted in PCA, revealing a spatial pattern. The analysis observed that the DS group clustered distinctly from the HC and nDS groups at PC1. Although the nDS and HC groups were distinct, many samples overlapped. These findings suggest qualitative and quantitative differences between the EV proteomics of the DS group compared to the others.

**FIGURE 3:**
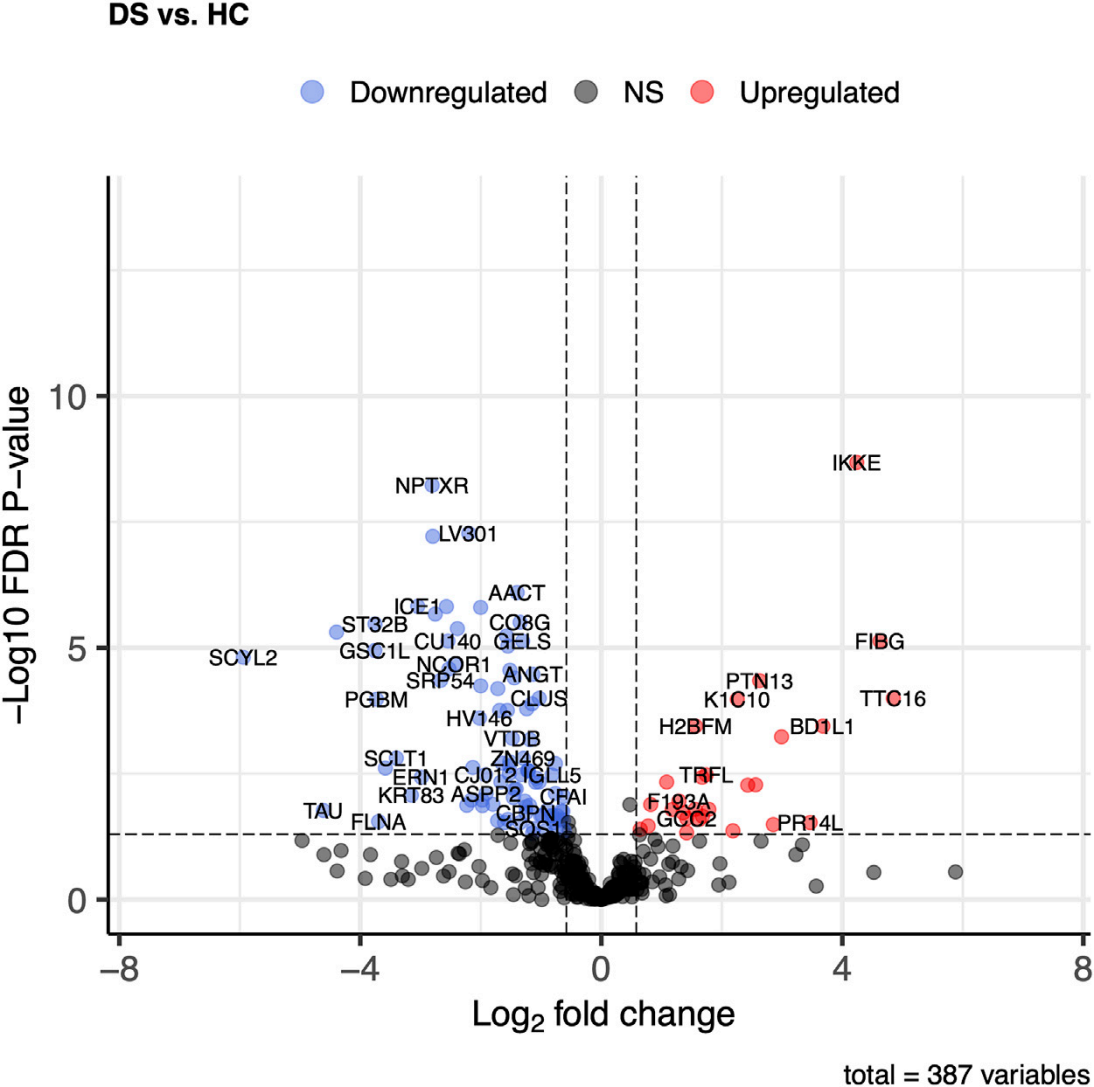
Comparison of proteome abundances in serum EVs of non-diabetic stroke (nDS) vs healthy control (HC). Volcano plots were used to compare protein concentrations at an adjusted *p* < 0.05 and log2FC ≥ 0.58 or log2FC ≤ -0.58.

Differential expression analysis of 387 proteins was performed to compare the nDS and HC groups using a general linear model. Herein nDS vs HC, only four proteins exhibited statistically significant changes (*p* ≤ 0.05 and log2FC ± 0.58). Comparing nDS with the HC group, only one protein (FIBG) was elevated in the nDS group, while three (FA5, PIGR, and TSP1) were higher in the HC group (Figure 3).

**FIGURE 4:**
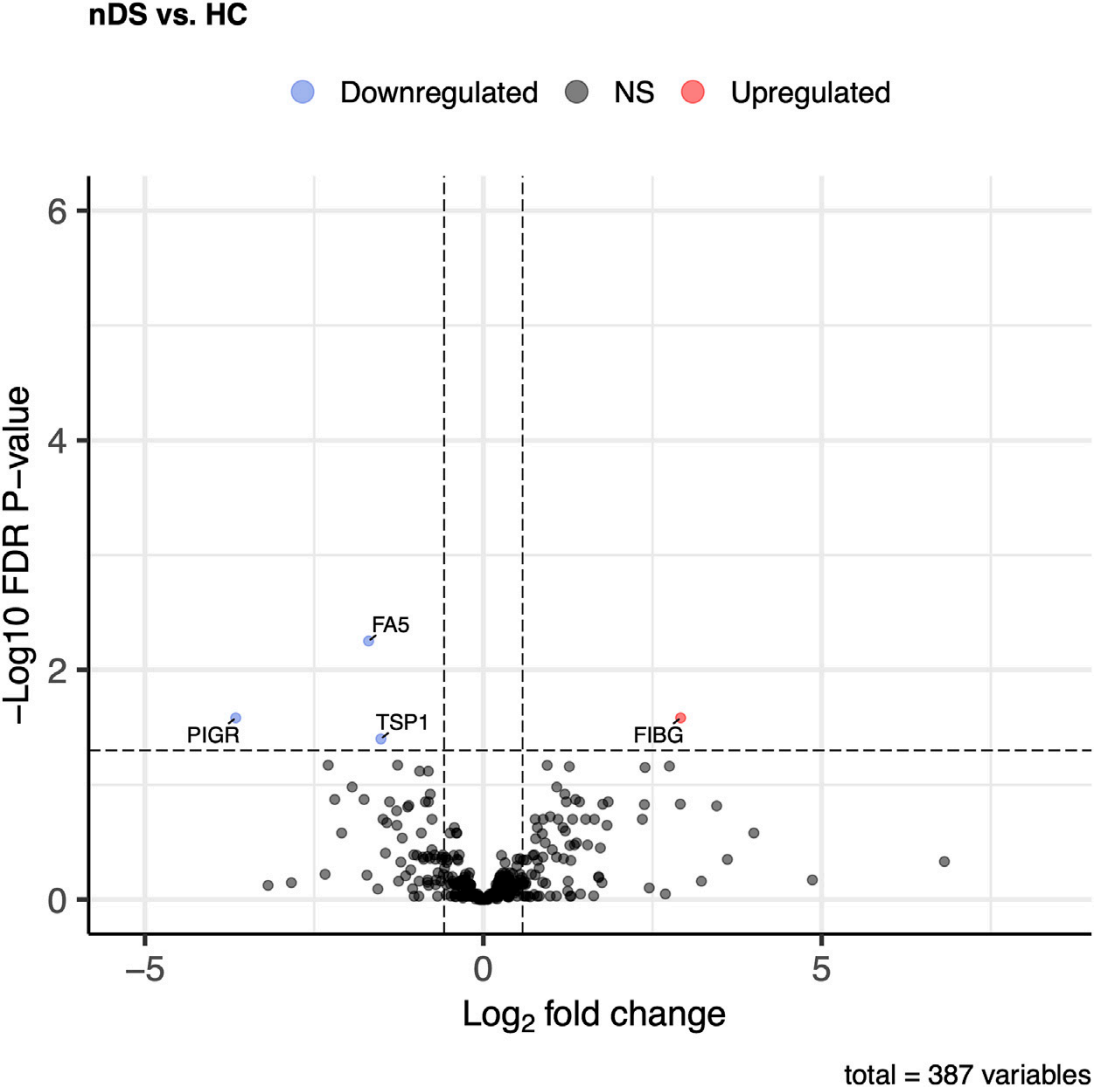
Comparison of proteome abundances in serum EVs of diabetic stroke (DS) vs healthy control (HC). Volcano plots were used to compare protein concentrations at an adjusted *p* < 0.05 and log2FC ≥ 0.58 or log2FC ≤ -0.58.

Of the DS vs HC, comparison of 387 proteins, 123 proteins exhibited significant changes (*p* ≤ 0.05 and log2FC ± 0.58). Twenty-eight proteins in the DS group showed increased levels compared to 95 proteins, which were higher in the HC group (Figure 4). The most elevated proteins in the DS group were TTC16, FIBG, IKKE, BD1L1, PR14L, and FIBB.

**FIGURE 5:**
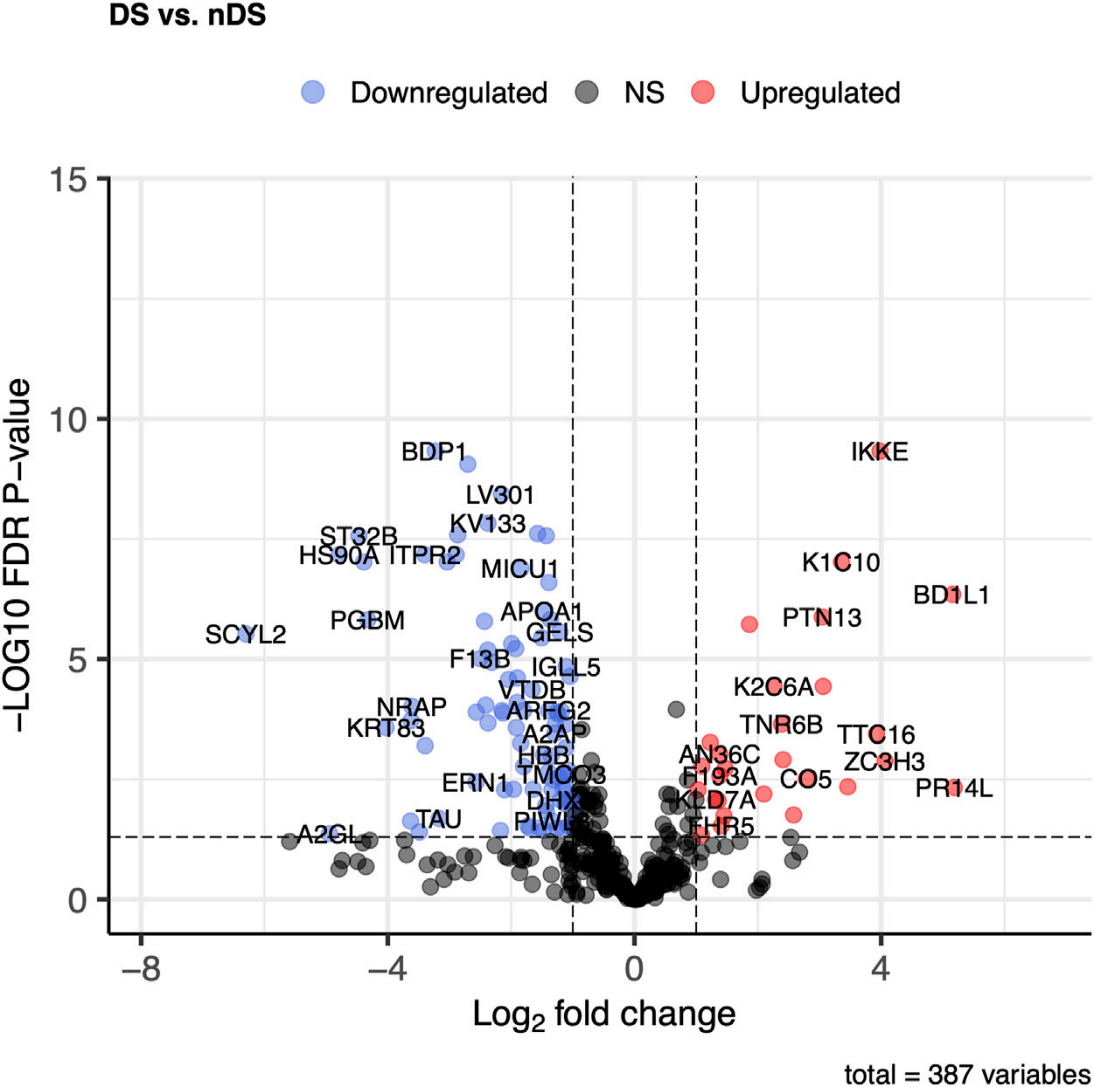
Comparison of proteome abundances in serum EVs of diabetic stroke (DS) vs. non-diabetic stroke (nDS). Volcano plots were used to compare protein concentrations at an adjusted *p* < 0.05 and log2FC ≥ 0.58 or log2FC ≤ -0.58.

The DS vs. nDS comparison found 149 proteins that differed significantly (*p* ≤ 0.05) and log2FC ±0.58). In the DS group, 33 proteins were elevated, while 116 were higher in the nDS group (Figure 5). The most elevated proteins in the DS group relative to the nDS group were PR14L, BD1L1, ZC3H3, IKKE, TTC16, DYH8, K1C10, and F13A.

### 3.4 Differential IPA analysis

**FIGURE 6:**
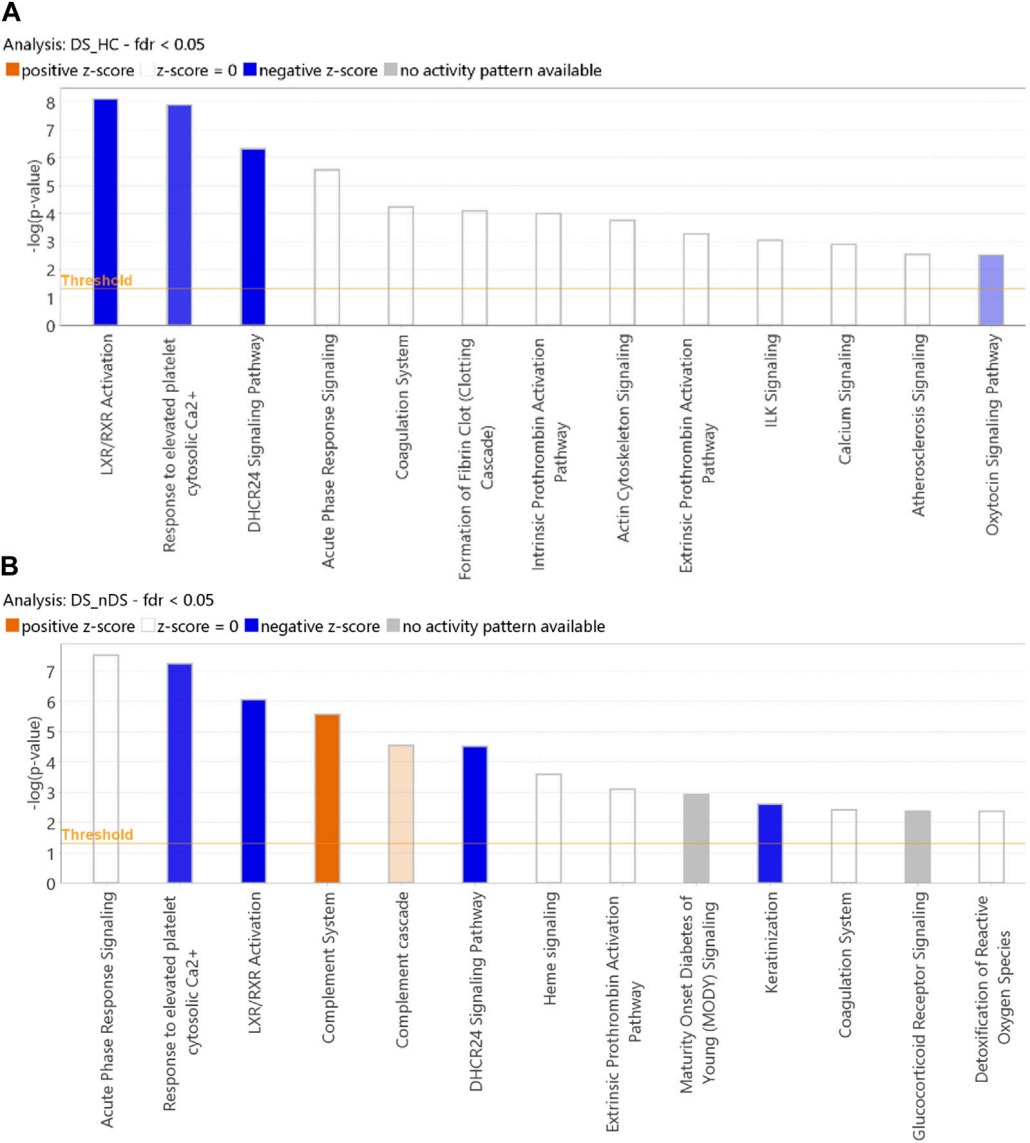
Classification of proteins by IPA core analysis. Bar charts show the classification of exosomal proteome measuring the likelihood of an association (Fisher’s Exact Test *p* ≤ 0.05) between the proteins and canonical pathway. The orange color bars in a canonical pathway represent the predicted activation (z-score >2), and the blue color bars represent the predicted inhibition (z-score ≤ -2). The grey bars display a z-score of NaN, representing that these pathways are ineligible for activity prediction. 2. White bars with a z-score = 0 represent that the similarities and dissimilarities between our dataset measurements and known information about the activity of the pathway balance out. **(A)** DS vs. HC analysis. **(B)** DS vs. nDS analysis.

IPA core function analysis investigates statistically significant (*p* ≤ 0.05) canonical pathways. Differentially expressed proteins (DS vs HC = 123 proteins and DS vs nDS = 149 proteins) were used for IPA core function analysis. Due to the presence of only four proteins with an FDR *p* ≤ 0.05 in the nDS vs HC comparison, conducting IPA pathways analysis for this comparison was not feasible. For DS vs HC results, “LXR/RXR activation” was the most inhibited pathway, followed by “response to elevated platelet cytosolic Ca^2+^“, and “DHCR24 signaling pathway” (Figure 6A). Nevertheless, several pathways, such as “acute response signaling,” “coagulation system,” and “fibrin clot formation,” exhibited notable activity, although discerning a specific pattern indicating the activation or inhibition of these pathways proved challenging. The IPA core analysis conducted on 149 differentially expressed proteins in the DS vs nDS comparison (Figure 6B) revealed the assignment of several pathways similar to those identified in the DS vs HC comparison. These pathways included “acute phase response signaling,” “LXR/RXR activation,” “response to elevated platelet cytosolic calcium,” and “DHCR24 signaling pathway.” Additionally, two pathways, “complement system” and “complement cascade,” were found to be activated.

## 4 Discussion

The popularity of exosomes resulted as a population of EVs, although International Society of Extracellular Vesicles (ISEV) 2018 concluded that the utilization of terminologies as exosomes or EVs is authors preference (Witwer and Théry, 2019). Nevertheless, EVs are primarily defined as bi-lipid layer vesicles released from cells irrespective of origin of lysosomal, multivesicular bodies (MVB), cell blebs and or micro-vesicles (Witwer and Théry, 2019). The cell blebs (apoptotic bodies) and microparticles are separated between 5000 and 20,000 g centrifugal force (Jeppesen et al., 2014). This study, isolated EVs by differential centrifugation, its last centrifugal fraction of EVs at 100,000 g is used in stroke biomarker study without any further classification based on endocytic origin. In general, EVs play a role in intercellular communication and disease pathogenesis, thus, EV proteomics in stroke research has the potential to discover novel biomarkers for disease diagnosis and recurrence prevention. Previous study of proteomic analysis demonstrated that EVs may be enriched biomarkers for new-onset stroke (Mitaki et al., 2021). The present study used high-performance mass spectrometry to analyse the proteome of EVs extracted from DS and nDS patients utilizing step gradient and ultracentrifugation technique to include all diverse populations of EVs. In total, 1,288 proteins were identified; however, 387 proteins with more than two peptides were statistically compared among the groups. The subsequent patterns of protein expression were generally associated with canonical pathways such as inhibition of LXR/RXR and coagulation and activation of complement system.

Functional enrichment analysis revealed that most proteins identified in the study were part of extracellular microparticles or organelles associated with the complement and coagulation cascades. PCA analysis of the proteome data revealed the most definite separation on PC1 based on sample groups. DS and nDS were distinctly clustered, while many samples from the HC group overlapped the nDS group, reflecting their similarity in protein expression. This distinct clustering pattern emphasizes the significance of diabetes as a prodromic or comorbid condition known to exacerbate stroke management (Amelia and Harahap, 2019).

Herein, we identified only four differentially expressed proteins in nDS vs HC comparison, out of which FIBG (Fibrinogen Gamma Chain) was the only protein over expressed in the nDS group. In contrast FA5 (Coagulation Factor V), PIGR (Polymeric Immunoglobulin Receptor), and TSP1 (Thrombospondin 1) were elevated proteins in the HC group.

These proteins play integral roles in the coagulation process, being released following vascular injury. Fibrinogen is an essential element of the coagulation system, and the increase in its concentrations is related to ischemic stroke. Fibrin/fibrinogen also represents a fundamental scaffolding component of thrombi retrieved by mechanical thrombectomy from ischemic stroke patients, as reported by structural studies (Moreno-Ramírez et al., 2018; Pir et al., 2022). Fibrins polymerize to form an insoluble fibrin matrix for blood clots; thus, its elevated levels are a risk factor for stroke. Thrombin cleaves fibrinogen to generate fibrin, the predominant constituent of blood clots. Hulshof et al., and Dagonnier et al., noted that fibrinogen and coagulation factors are pivotal in stroke pathogenesis, contributing to the coagulation cascade, further emphasizing the potential clinical application of fibrinogen in the acute stroke (Dagonnier et al., 2017; Hulshof et al., 2021). While the role of PIGR protein in stroke pathology remains poorly understood, Tromp et al. discovered that younger patients experiencing acute heart failure exhibited reduced levels of PIGR (Tromp et al., 2016). However, pathways and functional network analysis of these differentially expressed proteins revealed no evidence of pathway activation or inhibition in the nDS vs HC comparison.

Next, DS group in the DS vs. HC comparison showed twenty-eight elevated proteins, which are particularly linked to blood clotting. TTC16 (Tetratricopeptide repeat protein 16), FIBG, IKKE (Inhibitor of nuclear factor kappa-B kinase subunit epsilon), BD1L1 (Biorientation of chromosomes in cell division protein 1-like 1), PR14L (Proline Rich 14-Like Protein), and FIBB were the most differentially expressed proteins in the DS group, which demonstrated that EVs release the proteins of coagulation, structural, and anti-inflammatory pathways as an acute response to stroke. FIBG and FIBB polymerize to form an insoluble fibrin matrix for blood clots; thus, its elevated levels are a risk factor for stroke. TTC16 belongs to a family of structural tetratricopeptide repeat proteins that have shown changes in expression in ischemic stroke genome-wide association studies (Li, 2009; Moreno-Ramírez et al., 2018), which showed that anti-platelet therapy involving aspirin led to the downregulation of TTC16, and among several other proteins, in cardiovascular patients resistant to aspirin (Li, 2009). Notably, all patients in the DS group and 85% of patients in the nDS group were undergoing anti-platelet therapy, indicating the potential role of this protein in complications associated with stroke. Previously, an increase in CHD7 in circulating EVs has been reported as a novel endothelial dysfunction marker to monitor vascular condition in hypertensive patients (de la Cuesta et al., 2017). Similarly, mutation in CHD4 gene, a member of CHD subfamily, has been associated occlusive vascular disease, which leads to Moyamoya angiopathy and stroke (Pinard et al., 2020).

IKKE plays an essential role in innate immunity through NFkappaB, it also plays a role in platelets aggregation (Zhu et al., 2021). Recent studies show that IKK inhibitors could be potential antithrombotic agents (Karim et al., 2013). Therefore, higher levels of IKKE could indicate another possible cause of stroke, implying greater complexity in platelet activation pathways and a proclivity for severe disease outcomes in DS patients.

DS vs nDS comparison discovered 149 proteins, with many proteins following a pattern like DS vs. HC comparison. PR14L, BD1L1, ZC3H3, IKKE, TTC16, DYH8, K1C10, and F13A were the most differentially expressed proteins. PR14L, BD1L1, and ZC3H3 are the proteins that are not discussed above. Nevertheless, literature on these proteins association with stroke has not been found. These proteins are essential for chromosome biorientation and heterochromatin tethering, both of which support cell division (Poleshko et al., 2013; Higgs et al., 2015).

Further to proteins quantification data, similar trends were also observed for proteins associated pathway analysis for the DS vs. nDS comparisons. IPA core function analysis revealed that several pathways were over-activated or inhibited. The growing body of evidence suggests that the complement system is pivotal in the development of ischemic brain injury. Depleting specific complement components or inhibiting complement activation has been shown to mitigate ischemic brain injury (Ma et al., 2019). Therefore, in general, the complement system promotes inflammation, cerebral ischemic injury, and cell damage, contributing to stroke pathogenesis.

LXR/RXR activation was the most inhibited pathway in the DS group in both comparisons. The liver X receptors (LXRs) belong to the nuclear receptor superfamily and act as transcriptional regulators of cholesterol metabolism. LXR/RXR activation controls cholesterol homeostasis and inflammation in the brain, playing a potential protective role in atherosclerosis, infarction, and stroke pathogenesis (Courtney and Landreth, 2016) LXRs pair up with Retinoid X Receptors (RXRs), binding to specific DNA regions in gene promoters and enhancers, kicking off gene transcription. Coagulation pathways are defensive in preventing blood loss and debris accumulation at the injury site. Studies show that at the ischemic site, astrocytes, neurons, and microglia produce complement activation molecules (Arumugam et al., 2009).

Further discussion will delve into additional pathways that were inhibited in the DS group compared to both HC and nDS. Specifically, two pathways were notably inhibited: the “response to elevated platelet cytosolic Ca2+” pathway and the “DHCR24 signaling pathway.” The “response to elevated platelet cytosolic Ca2+” pathway, as also observed by Hu et al. (Hu et al., 2023) was found to be downregulated in ischemic stroke patients. This pathway is associated with platelet activation, signaling, and aggregation. Inhibition of this pathway may lead to a decrease in the release of platelet cytosolic Ca2+, potentially delaying platelet activation involved in hemostasis and thrombosis in stroke patients.

Similarly, in the DS group, the “DHCR24 signaling pathway” was inhibited, this pathway is known to have an anti-inflammatory neuroprotective role. Martiskainen et al. suggest that inhibition of this pathway could contribute to significant harm from stroke and inhibition of both the protective pathways in the DS group may result in a more severe response to stroke and delayed recovery (Martiskainen et al., 2017).

## 5 Conclusion

Herein we conclude that EVs carry a comprehensive proteome repertoire that can help elaborate disease-specific changes in diabetic stroke. Changes in proteome led to activation or inhibition of specific acute response pathways associated with tissue injury, inflammation, blood coagulation, and complement systems. Diabetes promotes coagulation and inflammation, which is linked to a poor prognosis after an acute ischemic stroke. These observations improve our understanding of the diverse regulatory functions of EVs in disease pathogenesis and provide new insight into targeting therapeutics in stroke prevention and post-stroke complications. Further studies are warranted with large sample size and targeted cardiovascular proteomics. In future, these stroke related biomarkers in EVs can be further distinguished by classifying them in EVs based on size compared to platelet derived microparticles/EVs for enhanced detection levels, minimizing the chance of serum contamination, and an insight of mechanistic approach in the development of therapeutic strategy for stroke.

## Data Availability

The mass spectrometry proteomics data have been deposited to the ProteomeXchange Consortium via the PRIDE partner repository with the dataset identifier PXD047674.
