# Supplementary material for: Mass spectrometry-based proteomic profiling of extracellular vesicle proteins in diabetic and non-diabetic ischemic stroke patients: a case-control study
Source: Front Mol Biosci. 2024 Jun 14;11:1387859. doi: 10.3389/fmolb.2024.1387859 (PMC11211575; doi:10.3389/fmolb.2024.1387859)

(A)

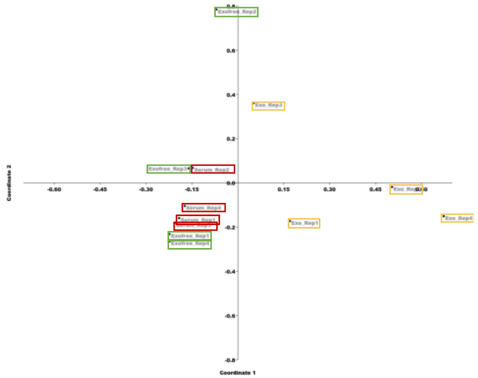

(B)

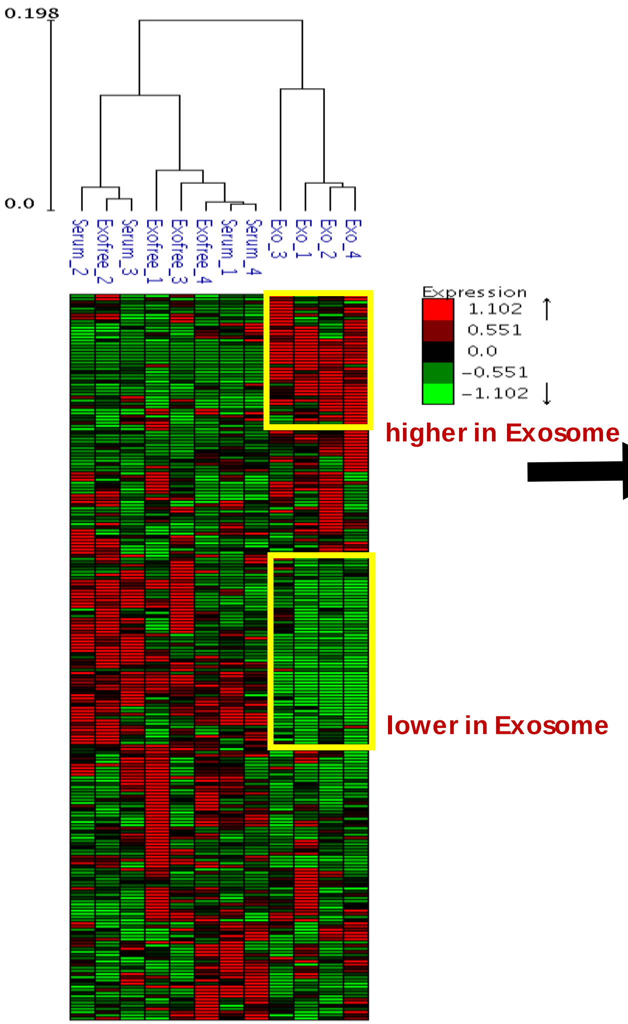

(C)

Protein_ID	p-value	U-score
HEL-214	0.0209	16.0
LGALS3BP	0.0209	16.0
IGHV3-72	0.0209	16.0
FCGBP	0.0209	16.0
TFRC	0.0209	16.0
IGJ;JCHAIN	0.0209	16.0
UNQ172;FCN3	0.0209	16.0
A2M	0.0209	16.0
C8B	0.0209	16.0
B4E1D8	0.0209	16.0
C1S	0.0209	16.0
C1R	0.0209	16.0
BCHE	0.0209	16.0
IGHM	0.0209	16.0
CD5L	0.0209	16.0
C1QC	0.0209	16.0
C1QB	0.0209	16.0
C1QA	0.0209	16.0
VWF	0.0209	16.0
V3-4;IGLV8-61	0.0209	16.0
PIGR	0.0209	16.0
Q86TT1	0.0209	16.0
PF4;PF4V1	0.0433	15.0
HPR	0.0433	15.0
C4BPB	0.0433	15.0
IGHV4-61	0.0433	15.0
HBD	0.0433	15.0
S6AWF47	0.0433	15.0
PROS1	0.0433	15.0
MASP1	0.194	12.5

(D)

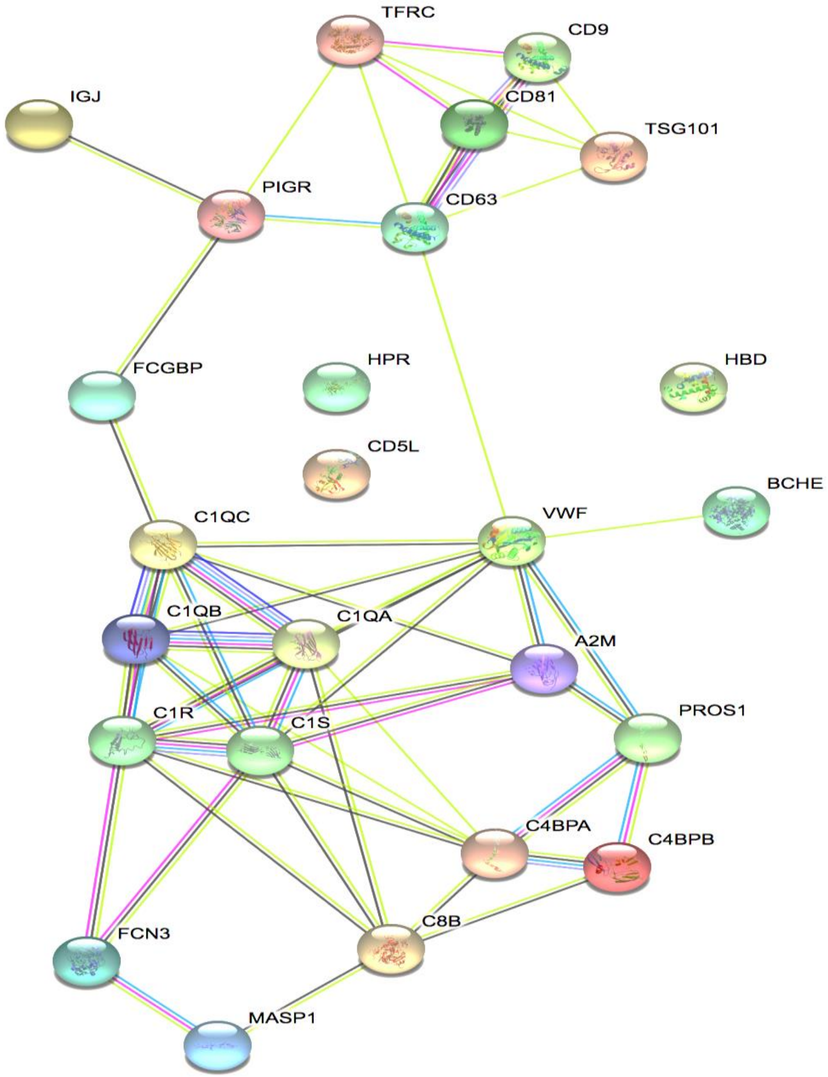

Supplement: Supplementary file 1 [file Image1.pdf]
